# Comparison of different target volume delineation strategies based on recurrence patterns in adjuvant radiotherapy for glioblastoma

**DOI:** 10.1093/nop/npae009

**Published:** 2024-01-31

**Authors:** Melek Tugce Yilmaz, Alper Kahvecioglu, Fazli Yagiz Yedekci, Ecem Yigit, Gokcen Coban Ciftci, Neyran Kertmen, Faruk Zorlu, Gozde Yazici

**Affiliations:** Department of Radiation Oncology, Faculty of Medicine, Hacettepe University, Ankara, Turkey; Department of Radiation Oncology, Faculty of Medicine, Hacettepe University, Ankara, Turkey; Department of Radiation Oncology, Faculty of Medicine, Hacettepe University, Ankara, Turkey; Department of Radiation Oncology, Faculty of Medicine, Hacettepe University, Ankara, Turkey; Radiology Department, Faculty of Medicine, Hacettepe University, Ankara, Turkey; Department of Medical Oncology, Faculty of Medicine, Hacettepe University, Ankara, Turkey; Department of Radiation Oncology, Faculty of Medicine, Hacettepe University, Ankara, Turkey; Department of Radiation Oncology, Faculty of Medicine, Hacettepe University, Ankara, Turkey

**Keywords:** clinical target volume, glioblastoma, radiotherapy, T2-FLAIR, target volume delineation

## Abstract

**Background:**

Radiation Therapy Oncology Group (RTOG) and the European Organization for Research and Treatment of Cancer (EORTC) recommendations are commonly used guidelines for adjuvant radiotherapy in glioblastoma. In our institutional protocol, we delineate T2-FLAIR alterations as gross target volume (GTV) with reduced clinical target volume (CTV) margins. We aimed to present our oncologic outcomes and compare the recurrence patterns and planning parameters with EORTC and RTOG delineation strategies.

**Methods:**

Eighty-one patients who received CRT between 2014 and 2021 were evaluated retrospectively. EORTC and RTOG delineations performed on the simulation computed tomography and recurrence patterns and planning parameters were compared between delineation strategies. Statistical Package for the Social Sciences (SPSS) version 23.0 (IBM, Armonk, NY, USA) was utilized for statistical analyses.

**Results:**

Median overall survival and progression-free survival were 21 months and 11 months, respectively. At a median 18 month follow-up, of the 48 patients for whom recurrence pattern analysis was performed, recurrence was encompassed by only our institutional protocol’s CTV in 13 (27%) of them. For the remaining 35 (73%) patients, recurrence was encompassed by all separate CTVs. In addition to the 100% rate of in-field recurrence, the smallest CTV and lower OAR doses were obtained by our protocol.

**Conclusions:**

The current study provides promising results for including the T2-FLAIR alterations to the GTV with smaller CTV margins with impressive survival outcomes without any marginal recurrence. The fact that our protocol did not result in larger irradiated brain volume is further encouraging in terms of toxicity.

Glioblastoma is the most common primary malignant central nervous system tumor in the adult population.^1^ Despite promising therapeutic improvements, the prognosis is dismal and the 5-year overall survival (OS) is less than 5%.^2^ Due to the infiltrative nature of the tumor, gross tumor resection (GTR) is challenging but has a survival advantage over subtotal tumor resection (STR).^3,4^ After pathological confirmation of glioblastoma, the standard of care is adjuvant chemoradiotherapy (CRT) with temozolomide, followed by adjuvant temozolomide.^5^ Patients who received adjuvant CRT have 2 times higher survival, as compared with the best supportive care.^6,7^

Although the role of radiotherapy (RT) in the treatment of glioblastoma is clear, there is still no consensus on target volume delineation. Recommendations from the Radiation Therapy Oncology Group (RTOG) and the European Organization for Research and Treatment of Cancer (EORTC) are commonly used guidelines.^8,9^ EORTC^9^ recommends one-phase irradiation as 60 Gy in 30 fractions and defines gross tumor volume (GTV) as T1 postcontrast (T1c+) enhancement area and resection cavity in post-operative magnetic resonance imaging (MRI) and excludes the peritumoral edema. GTV is expanded by 2–3 cm to obtain clinical target volume (CTV). RTOG^8^ on the other hand, suggests irradiating in 2 phases at 46 and 60 Gy. Phase-1 includes the T1c+ enhancement area, resection cavity, and hyperintensity in the T2-weighted and fluid-attenuated inversion recovery (FLAIR) with a 2 cm GTV1 to CTV1 margin. Phase-2 high-dose field includes the T1c+ enhancement area and resection cavity with also 2 cm GTV2 to CTV2 expansion. As an alternate target volume delineation strategy, The University of Texas MD Anderson Cancer Center (MDACC) utilizes a 2 cm margin around the GTV, which consists of the resection cavity and any residual contrast-enhancing tumor also ignoring the peritumoral edema.^10^ The main difference between target volume strategies comes from the decision of whether to include peritumoral edema in the treatment field.

There is no high benchmark standard regarding the inclusion of the T2-FLAIR non-contrast-enhancing alterations to the target volume. Several institutional series have shown results comparable to those of the literature when the T2-FLAIR alterations was not taken into account.^10,11^ However, significant tumor cell infiltration beyond the contrast-enhancing lesion and high cellularity across non-enhancing peritumoral tissue were demonstrated by pathology series.^12–14^ Hence, both T1c+ and T2-FLAIR sequences of MRI are used for GTV delineation in our institutional protocol and subsequent CTV is delineated by additional 0.5–1 cm margins to the GTV. In this study, we aimed to evaluate the oncological outcomes of patients treated with our institutional target volume delineation protocol and compare it with the RTOG and EORTC delineation strategies in terms of recurrence patterns and planning parameters.

## Materials and Methods

### Patient Population

**Figure 1.**
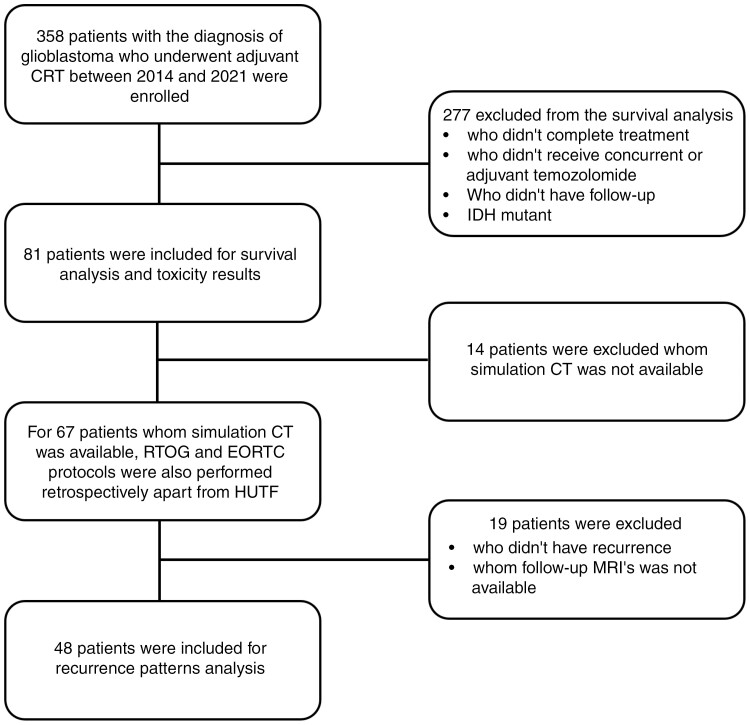
Consort flow chart. Abbreviations: CRT: chemoradiotherapy, IDH: isocitrate dehydrogenase, CT: computed tomograpy, MRI: magnetic resonance image.

A total of 358 patients with the diagnosis of glioblastoma who underwent adjuvant CRT in our department between the years of 2014 and 2021 were evaluated retrospectively. Patients who did not complete the intended treatment, did not receive concurrent or adjuvant temozolomide, did not receive 60 Gy in 30 fractions, and did not have appropriate follow-up imaging protocols in our center were excluded from the study. Considering the 2021 World Health Organization (WHO) classification, patients with isocitrate dehydrogenase (IDH) mutations were also excluded from the study.^15^ After the exclusions, a total of 81 patients were included (Figure 1). This retrospective study was conducted in compliance with the principles of the Helsinki Declaration and ethical approval was obtained from the institutional review board (GO 23/98, 2023/02-23).

### Target Volume Delineation

In our institutional target volume delineation protocol (CTV-HUTF), only one-phase irradiation of 60 Gy in 30 daily fractions is applied and it is founded on expert opinion and literature data that substantiates the presence of tumor cells in T2-FLAIR alterations. We include T2-FLAIR alterations into the GTV, with a reduced CTV margin, hence achieving a customized CTV for each individual patient. In order to clarify, the GTV was delineated as resection cavity + T2-FLAIR hyperintense areas ± residual tumor based on the both T1c+ and T2-FLAIR sequences of the MRI, which was performed within a maximum of one week prior to initiation of CRT. Subsequent CTV delineation is performed by an expansion of the GTV by 0.5–1 cm, excluding the critical structures. As for the critical structures, the CTV margin was 0 mm for the visual pathways, optic chiasm, and brainstem. Similarly, it was 0 mm for the skull and falx. However, for the ventricles, a set margin of 5 mm was imposed. In addition to our protocol, RTOG and EORTC protocols were also performed retrospectively in 67 patients (Figure 1). In the EORTC protocol (CTV-EORTC), GTV is delineated as resection cavity ± residual tumor based on the T1c + sequence of the MRI and CTV is delineated as GTV + 2.5 cm. In the RTOG protocol (CTV-RTOG), GTV-60 Gy is delineated as resection cavity ± residual tumor based on T1c + sequence of the MRI and CTV-60 Gy is delineated as GTV-60 + 2 cm. GTV-46 Gy is delineated as T2-FLAIR hyperintense regions + resection cavity ± residual tumor based on T1c+ and T2-FLAIR sequences of the MRI and CTV-46 Gy is delineated as GTV-46 + 2 cm. Planning target volume margins were 5 mm for all delineation protocols.

### Treatment and Follow-Up

The extent of resection is defined based on Response Assessment in Neuro-Oncology (RANO) criteria.^16^ Accordingly, GTR is defined as the removal of all contrast enhanced (CE) tumor or 95–99.9% resection of CE tumor and ≤1 cm^3^ residual CE tumor. STR is defined as 80–94.9% CE tumor reduction and ≤5 cm^3^ residual CE tumor or <80% CE tumor reduction and/or >5 cm^3^ residual CE tumor. After wound healing and removal of the metallic surgical clips, simulation CT (simCT) which has a 2.5 mm slice thickness was performed with intravenous contrast and a thermoplastic head mask for accurate immobilization. Sixty Gy of adjuvant CRT was applied to all patients in 30 fractions concurrent with 75 mg/m^2^ daily temozolomide. During CRT, all patients received trimethoprim-sulfamethoxazole at a dose of 800/160 mg twice a week for Pneumocystis Jirovecii prophylaxis. RT was applied via intensity-modulated radiotherapy (IMRT) or volumetric-modulated arc therapy via VersaHD® (Elekta AB, Stockholm, Sweden) or Clinac DHX® (Varian Medical Systems, Palo Alto, CA) devices. The hemogram and biochemistry parameters of the patients were checked weekly. After CRT, patients received adjuvant temozolomide for at least six cycles.

To follow up, a response evaluation was performed using brain MRI at 8th week after CRT, and repeated every 3 months after. Treatment response assessment was performed using the RANO criteria.^17^ Progression is defined as a ≥25% rise in the total of the products of the perpendicular diameters of contrast-enhancing lesions or any new lesion. In patients with suspected pseudo-progression, progression was verified by MRI spectroscopy.

### Recurrence Pattern Analysis

In 48 of the 60 locally-recurrent patients, MRI imaging that detected local recurrence (LR) could be fused with simCT images and the recurrence was delineated as GTV-recurrence (Figure 1). After the fusion and delineation of different CTVs, all recurrences were evaluated in terms of localization on the 3 different CTV contours (CTV-HUTF, CTV-EORTC, and CTV-RTOG). If the GTV-recurrence was encompassed by CTV completely, it was considered an in-field recurrence but if the GTV-recurrence has components both in and out of the CTV, recurrence was considered as marginal.

The positional differences between the centers of 3 different CTV definitions and the center of the GTV-recurrence volume were measured in each of the 48 recurrent patients. The median distance between the centers was calculated. In addition, to mathematically quantify the relationship between CTV volumes and recurrent volumes, we performed a calculation in which the GTV-recurrence volumes that were confined within the CTV were divided by the total GTV-recurrence volume. This operation resulted in a ratio that ranges from 0 to 1, where a value of 1 denotes that the GTV-recurrent volume is completely encapsulated within the CTV volume. Conversely, a value of 0 indicates that the GTV-recurrence volume is completely outside of the CTV volume. A value between 0 and 1 represents the proportional percentage of the GTV-recurrence volume that is located within the confines of the CTV volume.

### Statistical Analysis

All time-related events were defined from the diagnosis to the last follow-up, death, or recurrence, whichever came first. Kaplan–Meier method was used for survival analysis and the log-rank test for comparison. Variables that were significant on univariate analysis were included in the multivariate models. Student *t*-test was used to compare mean distances between recurrence and CTV centers and to compare target volumes, organs at risk volume and doses between 3 delineation protocols Kruskal–Wallis tests were conducted. The Mann–Whitney U test was performed to test the pairwise differences using Bonferroni correction. Statistical Package for the Social Sciences (SPSS) version 23.0 (IBM, Armonk, NY, USA) was utilized for all statistical analyses, including descriptive, OS, and, progression-free survival (PFS). A *P*-value <.05 was considered statistically significant.

## Results

### Patient, Tumor, and Treatment Characteristics

The baseline patient, tumor, and treatment characteristics are summarized in Table 1. All patients underwent a tumor resection before RT. GTR was performed in 23 (%28.4) and STR was performed in 58 (%71.6) patients. The time interval between surgery and the first fraction of RT was a median of 26 days (range, 15–35 days). None of the patients had undergone a second look surgery before CRT.

**Table 1.** Patient, Tumor and Treatment Characteristics

Characteristic	Number of patients (%)
Age (Median, range)	55 years (20–71 years)
Gender • Male • Female	45 (55.6%) 36 (44.4%)
RT schedule	60 Gy/30 fractions
Concurrent temozolomide dose	75 mg/m^2^, d1–7 q1 week
Adjuvant temozolomide dose	150 to 200 mg/m^2^, d1–5 q4 weeks × 6–12 cycles
Adjuvant temozolomide duration • 6 months • 12 months • Unknown duration	34 (42%) 26 (32.1%) 21 (25.9%)

Abbreviations: RT = radiotherapy.

### Treatment Outcomes and Prognostic Factors

The median follow-up period was 18 months (range, 4–69 months). During follow-up, LR was observed in 60 patients (74.1%). The 1- and 2-year OS and PFS rates were 78% and 43% and, 47% and 22%, respectively. The median OS was 21 months (standard error [SE]:2.64; 95% confidence interval [CI]: 16.1–26.4) and the median PFS was 11 months (SE:1.4; 95% CI: 8.7–14.4).

In univariate analysis, CTV volume (<207 cc vs. ≥207 cc) was prognostic for OS (*P* =.03) and age (<50 vs. ≥50 years) was determined as borderline significant (*P* = .09). None of the parameters were prognostic for PFS. Multivariate Cox regression analysis was performed to identify the factors correlating with OS and age of the patient (hazard ratio [HR] 0.483; 95% CI, 0.248–941; *P* = .03) and CTV volume were found to be significant independent factors correlating with OS (HR: 0.461; 95% CI: 0.243–0.874; *P* = .01).

### Comparison of Recurrence Patterns

**Figure 2.**
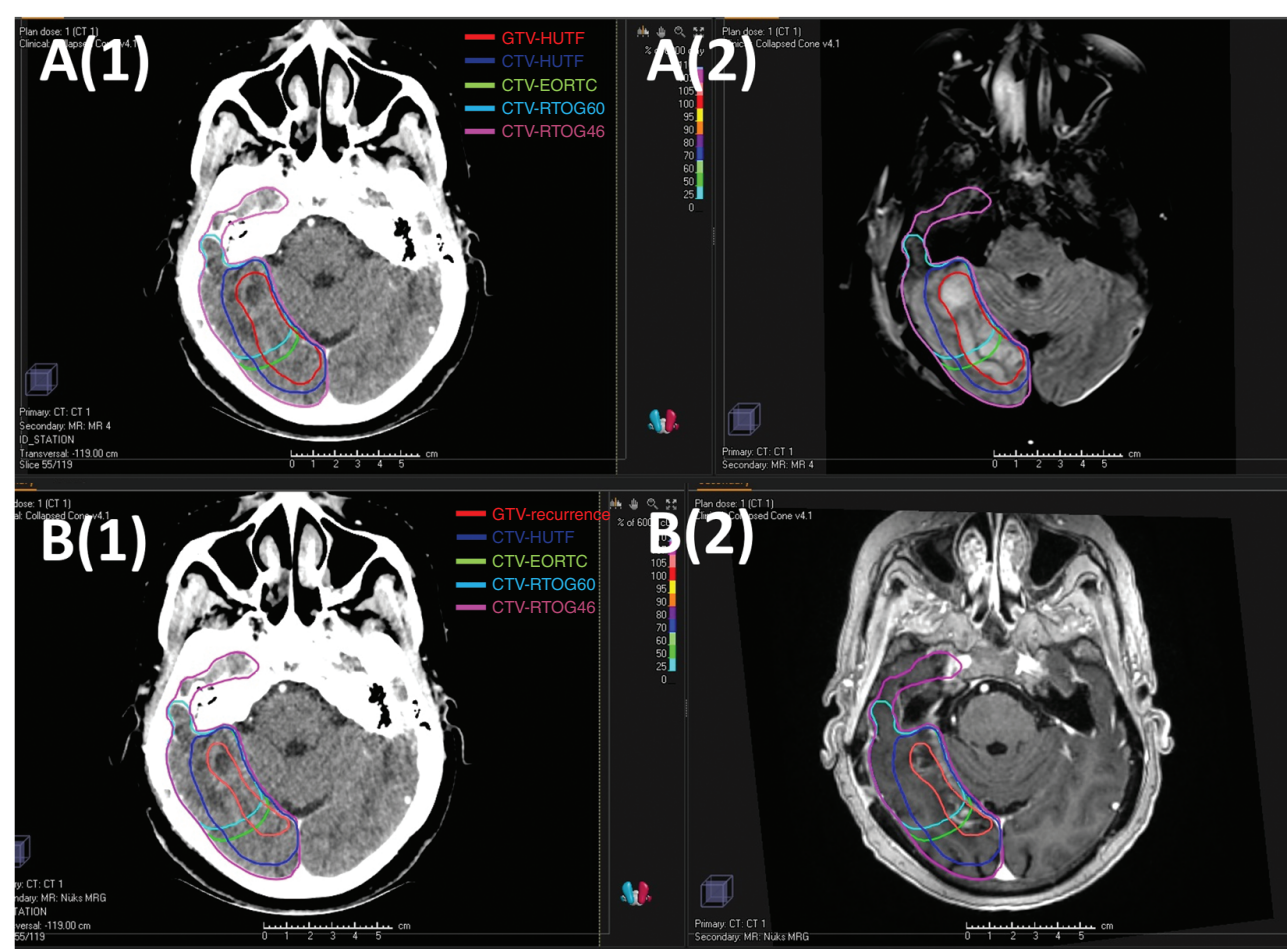
Recurrence pattern analysis for different treatment target volume delineation strategies. (A) Axial images of the simCT (1) and T2-FLAIR sequence of post-operative MRI (2). The red line is our institutional protocol’s GTV including both resection cavity and T2-FLAIR alterations. Dark blue line is CTV-HUTF (GTV + 1 cm). The green line represents CTV-EORTC, the light blue line represents CTV-RTOG60 and the pink represents the CTV-RTOG46. The volumes of each CTV for EORTC, RTOG, and CTV-HUTF are CTV 194 cc, 168 cc, and 171 cc, respectively. (B) Axial images of the simCT (1) and T1c + sequence of MRI (2) which has detected the LR. The red line is GTV-recurrence. The dark blue line is CTV-HUTF and green line represents CTV-EORTC. The dark blue line is CTV-RTOG60 and the pink line is CTV-RTOG46. As shown in the figure, recurrent lesion occurred in the previous T2-FLAIR weighted region and were encompassed by CTV-HUTF only. For EORTC and RTOG protocols, the recurrence is marginal. Abbreviations: simCT: simulation computed tomography, FLAIR: fluid-attenuated inversion recovery, MRI = magnetic resonance imaging, GTV: gross tumor volume, CTV = clinically target volume, EORTC = European organization for research and treatment of cancer, RTOG = radiation therapy oncology group, T1c+ = t1 weighted postcontrast.

Of the 60 patients with LR, 48 of them had RTOG, EORTC, and our institutional delineation protocol delineated and could be compared (Figure 1). In 13 patients (%27), GTV-recurrence was encompassed by only CTV-HUTF and marginal recurrence was observed in the CTV-EORTC and CTV-RTOG (Figure 2). For the remaining 35 locally recurred patients (%73), recurrence was developed directly from the resection cavity or post-operative residual tumor and the GTV-recurrence was within all 3 separate CTVs (Table 2). There was no marginal recurrence in our delineation protocol and all LRs were considered as an in-field recurrence.

**Table 2.** Comparison of Recurrence Patterns

Protocol name	In-field recurrence (%)	Marginal recurrence	Distant recurrence
RTOG60	35 (73%)	13 (27%)	–
EORTC	35 (73%)	13 (27%)	–
HUTF	48 (100%)	–	–

Abbreviations: RTOG: Radiation Therapy Oncology Group, EORTC: European Organization for Research and Treatment of Cancer, HUTF: Hacettepe University Medical School.

The volumetric relationship between recurrence volumes and CTVs was examined, revealing that the score was consistently 1 for all CTV-HUTF cases. However, in a subset of 13 patients whose recurrence was only encompassed by CTV-HUTF, this score deviated from 1 for CTV-EORTC, CTV-RTOG46, and CTV-RTOG60. Specifically, the CTV-EORTC score ranged from 0.65 to 0.94 in these patients, while the scores for CTV-RTOG46 and CTV-RTOG60 ranged from 0.55 to 0.93. Based on our findings, median distances between the isocenter of GTV-recurrence and the isocenters of CTV-HUTF, CTV-EORTC, CTV-RTOG46, and CTV-RTOG60 were 0.61 (range, 0–1.75), 0.75 (range, 0.07–2), 0.75 (range, 0-2.75), and 0.50 (range, 0–1.75) cm, respectively. The distances between the isocenters of CTV-HUTF and CTV-EORTC and the GTV-recurrence isocenter were not statistically significant (*P* = .26). However, the distance between the isocenter of CTV-RTOG46 and the GTV-recurrence isocenter was greater than that of CTV-HUTF (*P* < .001). On the other hand, the distance between the isocenter of CTV-RTOG60 and the GTV-recurrence isocenter was smaller than that of CTV-HUTF (*P* < .001).

### Planning Parameters

A comparison of target volumes, OAR volume, and doses of different target volume delineation protocols was presented in Table 3. Our GTV was higher than both RTOG and EORTC GTVs as we delineated both contrast-enhanced lesions and T2-FLAIR (*P* < .001). As for CTV volumes, CTV-HUTF was smaller than CTV-EORTC (*P* < .001) but there was no difference between CTV-HUTF and CTV-RTOG60 Gy (*P* = .4). The brain volume outside CTV was calculated and our protocol had a larger unirradiated brain volume than CTV-EORTC and CTV-RTOG46 (*P* = .04 and *P* < .001).

**Table 3.** Comparison of Planning Parameters Between Different Target Volume Delineation Protocols

	EORTC protocol	RTOG protocol (60 Gy)	RTOG protocol (46 Gy)	HUTF protocol	*P-*value^*^
GTV (median)	43 cc (range, 6–189 cc)	43 cc (range, 6–189 cc)	107 cc (range, 14–259 cc)	107 cc (range, 14–259 cc)	<.001
CTV (median)	275 cc (range, 125–601 cc)	212 cc (range, 93–521 cc)	357 cc (range, 157–594 cc)	211 cc (range, 44–475 cc)	<.001
Brain volume outside CTV (median)	1131 cc (range, 811–1571 cc)	1186 cc (range, 863–1603 cc)	1025 cc (range, 712–1600 cc)	1187 cc (range, 809–1669 cc)	<0.001
Mean brain dose (Median, range)	3149 cGy (range, 1570–4430)	3503 (range, 2080–4930)	3503 (range, 2080–4930)	2832 cGy (range, 1340–4160)	<.001
Brain V40 (median, range)	33% (range, 11–67%)	41% (range, 17–61%)	41.5% (range, 17–61%)	29% (range, 7–55%)	<.001
Brain V30 (median, range)	42% (range, 19–76%)	53% (range, 23–77%)	53% (range, 23–77%)	40% (range, 12–68%)	<.001
Brain V20 (median, range)	66% (range, 27–90%)	73% (range, 35–90%)	73% (range, 35–90%)	59% (range, 20–87%)	<.001
Mean left hippocampi dose (median, range)	3535 cGy (range, 128–6095 cGy)	4025 cGy (range, 583–6090 cGy)	4025 cGy (range, 583–6090 cGy)	2970 cGy (range, 108–6070 cGy)	.02
Mean right hippocampi dose (median, range)	3935 cGy (range, 740–6520 cGy)	4001 cGy (range, 984–6080 cGy)	4001 cGy (range, 984–6080 cGy)	3290 cGy (range, 167–6080 cGy)	.07

Abbreviations: GTV = gross tumor volume, CTV = clinical target volume, EORTC = European Organization for Research and Treatment of Cancer, RTOG = Radiation Therapy Oncology Group, *V*_x_ = volume of receiving ≥ X Gy.

^*^Kruskal–Wallis Test conducted to compare 4 different protocols.

As for OAR doses, mean brain dose were lower in CTV-HUTF than both CTV-EORTC and CTV-RTOG (*P* = .002 and *P* < .001, respectively). Again, brain V40, V30, and V20 doses were lower in our protocol (*P* < .001, *P* < .001, *P* < .001). Hippocampal dosimetric parameters were also calculated and CTV-HUTF left hippocampus mean dose was lower than the RTOG protocol (*P* = .01).

### Toxicity

Treatment related toxicities were evaluated for 80 of the 81 patients. The most common grade 1 or 2 acute toxicity was nausea (*n* = 57, 70%) followed by headache (*n* = 54, 66%) and mild cytopenia (*n* = 26, 32%). None of the patients experienced severe (≥grade 3) hematological or neurological acute or late toxicity.

## Discussion

In our study, we observed that target volume delineation by including the T2-FLAIR to the GTV and using smaller margins (ie, 0.5–1 cm) for CTV is associated with excellent coverage of the recurrence and causes less normal brain tissue irradiation compared to the EORTC and RTOG protocols. The absence of marginal recurrence despite the reduced CTV margins implies that a case-based 0.5–1 cm margin is sufficient in cases where T2-FLAIR is included in the GTV. In addition, our median 21 months OS and 11 months PFS exhibit notable improvements compared to the historical series.^18^ These survival outcomes are also one of the longest in contemporary series.^11,19–21^ Similar to our study, Duma et al.^19^ reported 23-months median survival in their leading-edge radiosurgery technique which they delivered a median of 8 Gy to the T2-FLAIR regions before or during CRT. Uncoincidentally, these 2 publications irradiating T2-FLAIR achieved similar elongated survival outcomes, providing evidence for investigating the inclusion of T2-FLAIR alterations to the RT field.

One of the key research topics in glioblastoma is that the T2-FLAIR signals in the vicinity of the contrast-enhanced lesion may contain tumor cells.^12–14^ Tumor cells were often discovered at least 1 cm beyond the tumor edge, according to Yamahara et al.^12^ who also noted that bigger tumor edema in the T2 series displayed a larger infiltrative pattern. Moreover, Barajas et al.^13^ showed that 81% of the samples collected from noncontrast-enhancing lesions had tumor cells. Glioblastoma’s infiltrative nature poses a challenge for the tumor removal with larger margins meaning supra-total resection.^22^ Institutional retrospective studies demonstrate that performing a “FLAIRectomy” during surgery, which involves removing the FLAIR and the contrast-enhancing lesion, improves OS.^23–26^ In MDACC’s cohort of 1229 patients, cases with removal of >53% of FLAIR changes were associated with prolonged OS.^24^ Further, in the recently published RANO resect group study, “supramaximal resection” which is defined as the removal of the T2/FLAIR abnormalities was associated with improved OS.^16^ However, the greatest drawback of supramaximal resection is the greater likelihood of morbidity. Patients with glioblastoma who experience post-surgical morbidity have worse survival rates.^27,28^ Maintaining the delicate balance between post-operative morbidity and supramaximal resection appears to be crucial.

The fundamental issue with the incorporation of T2-FLAIR alterations into the RT field is the fact that these FLAIR signals are not specific to tumor infiltration and also represent vasogenic edema, gliosis, inflammation, or post-operative ischemia.^29^ Also, anti-edema treatments and the timing of the MRI scan could affect T2-FLAIR volume.^30^ Although MRI features like gray matter involvement, FLAIR hyperintensity density, and the presence of diffuse mass effect are suggested in the differentiation of inflammation vs. tumor infiltration, It is not possible to make a clear distinction.^31^ Additionally, the inclusion of T2-FLAIR in the RT field raises concerns about the enlargement of treatment volumes and the consequent increase in morbidity. The decrease in cognitive functions due to RT increases with dose and volume.^32^ Also, hippocampus dose is important in terms of glial neurogenesis and protection of long-term cognitive function.^33,34^ Although the volume of GTV increased in our study due to the inclusion of FLAIR; median CTV volume, mean and V20–V30–V40 brain doses and mean hippocampus doses were the lowest compared to the EORTC and RTOG protocols. This is proof that our way of FLAIR-irradiation with a modified CTV does not cause the large treatment fields. Also, our results indicate that treatment volume is associated with OS compatible with the literature.^10^

Numerous retrospective and prospective studies delved into the impact of smaller CTV margins on patient survival and the pattern of recurrence in glioblastoma.^10,11,35–40^ Especially the fact that 80–90% of the recurrences occur around the GTV and that intra-field recurrences are inevitable even in studies where the dose is up to 70–90 Gy are the strongest arguments for reducing the margins.^41–44^ According to Langhans et al.^40^ the reduced margin is feasible and that anisotropic margins given using radiomics may be a better option for treatment. Minniti et al.^39^ also reported that there was no increase in marginal recurrence with 1 cm compared to EORTC delineation, but an increase of 0.5 cm was observed. We also used similar reduced margins in our study and even further deescalated the margin to 0.5–1 cm. However, a major difference between the aforementioned studies from ours is that they did not include the T2-FLAIR abnormalities in their CTV. Further, the reduced CTV given to the T2-FLAIR alterations in our study can be considered as one of the early examples of the reduced anisotropic margin suggested by Langhans et al.^40^ The current ESTRO-EANO guideline recommends a GTV to CTV margin of 1.5 cm^3^. It is also stated that the T2-FLAIR signal changes are likely to represent the tumor and should be included in the treatment volume especially non-contrast enhanced tumor is suspected, and in that case margin of 0–15 mm is recommended even though no consensus could be reached. The margins selected in our study seem to be compatible with current guidelines.

Although we report excellent survival outcomes with zero marginal recurrences despite smaller target volumes, there are certain limitations. First, the results’ generalizability is limited due to the retrospective methodology and in order to assess the efficacy of our protocol, we included patients who had successfully completed the treatment protocol. However, it is important to acknowledge that this approach may have introduced potential selection bias. The second potential drawback of our study is that, although smaller target volumes and lower OAR doses may result in potentially lower rates of toxicity, the relevance of these results in terms of clinical toxicity outcomes is not known. Also, missing data is another limitation in reducing the number of patients. Last but not least, the delineation protocol that we used in our study can be further improved. MRI spectroscopy or FLAIR apparent diffusion coefficient map can be used to distinguish the non-enhanced tumor from edema.^45^ Also, amino acid radiolabeled metabolic positron emission tomography (PET) and PET/MRI seem to be very promising.^46,47^ Thus, there may be a chance to further de-escalate the treatment volume.

## Conclusions

In patients with glioblastoma, most of the LRs after adjuvant CRT ± adjuvant chemotherapy occur in the resection cavity, residual tumor region, or within 1 cm around the T2-FLAIR alterations. Inclusion of the peritumoral T2-FLAIR signal changes to the GTV and delineation of the CTV by a 0.5–1 cm margin is associated with decreased OAR doses compared with the RTOG and EORTC protocols, without any marginal recurrence. Hypothetically, it seems that the treatment of glioblastoma in the future will be based on the upfront “*neurocognitive function-sparing supramaximal resection”* with an adjuvant “*FLA-IR-radiation CRT*.” However, the role of different delineation protocols warrants further prospective investigation, focusing on recurrence patterns and toxicity for deciding on the optimal delineation approach.

## Data sharing

The datasets generated and analyzed during the current study are not publicly available but are available from the corresponding author on reasonable request.

## References

[1] Ostrom QT , Price M, Neff C, et al. CBTRUS statistical report: primary brain and other central nervous system tumors diagnosed in the United States in 2015–2019. Neuro Oncol. 2022;24(Suppl 5):v1–v95. 36196752 10.1093/neuonc/noac202 PMC9533228

[2] Koshy M , Villano JL, Dolecek TA, et al. Improved survival time trends for glioblastoma using the SEER 17 population-based registries. J Neurooncol. 2012;107(1):207–212. 21984115 10.1007/s11060-011-0738-7 PMC4077033

[3] Lacroix M , Abi-Said D, Fourney DR, et al. A multivariate analysis of 416 patients with glioblastoma multiforme: prognosis, extent of resection, and survival. J Neurosurg. 2001;95(2):190–198. 10.3171/jns.2001.95.2.0190 11780887

[4] Brown TJ , Brennan MC, Li M, et al. Association of the extent of resection with survival in glioblastoma: a systematic review and meta-analysis. JAMA Oncol. 2016;2(11):1460–1469. 27310651 10.1001/jamaoncol.2016.1373 PMC6438173

[5] Stupp R , Hegi ME, Mason WP, et al; European Organisation for Research and Treatment of Cancer Brain Tumour and Radiation Oncology Groups. Effects of radiotherapy with concomitant and adjuvant temozolomide versus radiotherapy alone on survival in glioblastoma in a randomised phase III study: 5-year analysis of the EORTC-NCIC trial. Lancet Oncol. 2009;10(5):459–466. 19269895 10.1016/S1470-2045(09)70025-7

[6] Walker MD , Alexander E, Jr, Hunt WE, et al. Evaluation of BCNU and/or radiotherapy in the treatment of anaplastic gliomas. A cooperative clinical trial. J Neurosurg. 1978;49(3):333–343. 355604 10.3171/jns.1978.49.3.0333

[7] Kristiansen K , Hagen S, Kollevold T, et al. Combined modality therapy of operated astrocytomas grade III and IV. Confirmation of the value of postoperative irradiation and lack of potentiation of bleomycin on survival time: a prospective multicenter trial of the Scandinavian Glioblastoma Study Group. Cancer. 1981;47(4):649–652. 6164465 10.1002/1097-0142(19810215)47:4<649::aid-cncr2820470405>3.0.co;2-w

[8] Cabrera AR , Kirkpatrick JP, Fiveash JB, et al. Radiation therapy for glioblastoma: executive summary of an American Society for Radiation Oncology Evidence-Based Clinical Practice Guideline. Pract Radiat Oncol. 2016;6(4):217–225. 27211230 10.1016/j.prro.2016.03.007

[9] Niyazi M , Brada M, Chalmers AJ, et al. ESTRO-ACROP guideline “target delineation of glioblastomas.”. Radiother Oncol. 2016;118(1):35–42. 26777122 10.1016/j.radonc.2015.12.003

[10] Kumar N , Kumar R, Sharma SC, et al. Impact of volume of irradiation on survival and quality of life in glioblastoma: a prospective, phase 2, randomized comparison of RTOG and MDACC protocols. Neurooncol Pract. 2020;7(1):86–93. 32257287 10.1093/nop/npz024 PMC7104885

[11] Zheng L , Zhou ZR, Yu Q, et al. The definition and delineation of the target area of radiotherapy based on the recurrence pattern of glioblastoma after temozolomide chemoradiotherapy. Front Oncol. 2020;10:615368. 33692942 10.3389/fonc.2020.615368 PMC7937883

[12] Yamahara T , Numa Y, Oishi T, et al. Morphological and flow cytometric analysis of cell infiltration in glioblastoma: a comparison of autopsy brain and neuroimaging. Brain Tumor Pathol. 2010;27(2):81–87. 21046309 10.1007/s10014-010-0275-7

[13] Barajas RF, Jr, Phillips JJ, Parvataneni R, et al. Regional variation in histopathologic features of tumor specimens from treatment-naive glioblastoma correlates with anatomic and physiologic MR Imaging. Neuro Oncol. 2012;14(7):942–954. 22711606 10.1093/neuonc/nos128 PMC3379808

[14] Eidel O , Burth S, Neumann JO, et al. Tumor infiltration in enhancing and non-enhancing parts of glioblastoma: a correlation with histopathology. PLoS One. 2017;12(1):e0169292. 28103256 10.1371/journal.pone.0169292 PMC5245878

[15] Louis DN , Perry A, Wesseling P, et al. The 2021 WHO classification of tumors of the central nervous system: a summary. Neuro Oncol. 2021;23(8):1231–1251. 34185076 10.1093/neuonc/noab106 PMC8328013

[16] Karschnia P , Young JS, Dono A, et al. Prognostic validation of a new classification system for extent of resection in glioblastoma: a report of the RANO resect group. Neuro Oncol. 2023;25(5):940–954. 35961053 10.1093/neuonc/noac193 PMC10158281

[17] Wen PY , Macdonald DR, Reardon DA, et al. Updated response assessment criteria for high-grade gliomas: response assessment in neuro-oncology working group. J Clin Oncol. 2010;28(11):1963–1972. 20231676 10.1200/JCO.2009.26.3541

[18] Stupp R , Mason WP, van den Bent MJ, et al; European Organisation for Research and Treatment of Cancer Brain Tumor and Radiotherapy Groups. Radiotherapy plus concomitant and adjuvant temozolomide for glioblastoma. N Engl J Med. 2005;352(10):987–996. 15758009 10.1056/NEJMoa043330

[19] Duma CM , Kim BS, Chen PV, et al. Upfront boost Gamma Knife “leading-edge” radiosurgery to FLAIR MRI-defined tumor migration pathways in 174 patients with glioblastoma multiforme: a 15-year assessment of a novel therapy. J Neurosurg. 2016;125(Suppl 1):40–49. 27903197 10.3171/2016.7.GKS161460

[20] Brown PD , Chung C, Liu DD, et al. A prospective phase II randomized trial of proton radiotherapy vs intensity-modulated radiotherapy for patients with newly diagnosed glioblastoma. Neuro-Oncol. 2021;23(8):1337–1347. 33647972 10.1093/neuonc/noab040 PMC8328012

[21] Omuro A , Brandes AA, Carpentier AF, et al. Radiotherapy combined with nivolumab or temozolomide for newly diagnosed glioblastoma with unmethylated MGMT promoter: An international randomized phase III trial. Neuro Oncol. 2023;25(1):123–134. 35419607 10.1093/neuonc/noac099 PMC9825306

[22] Haddad AF , Young JS, Morshed RA, Berger MS. FLAIRectomy: resecting beyond the Contrast Margin for Glioblastoma. Brain Sci. 2022;12(5):544–554. 35624931 10.3390/brainsci12050544 PMC9139350

[23] Sanai N , Polley MY, McDermott MW, Parsa AT, Berger MS. An extent of resection threshold for newly diagnosed glioblastomas. J Neurosurg. 2011;115(1):3–8. 21417701 10.3171/2011.2.jns10998

[24] Li YM , Suki D, Hess K, Sawaya R. The influence of maximum safe resection of glioblastoma on survival in 1229 patients: Can we do better than gross-total resection? J Neurosurg. 2016;124(4):977–988. 26495941 10.3171/2015.5.JNS142087

[25] Vivas-Buitrago T , Domingo RA, Tripathi S, et al. Influence of supramarginal resection on survival outcomes after gross-total resection of IDH–wild-type glioblastoma. J Neurosurg. 2022;136(1):1–8. 10.3171/2020.10.JNS203366 PMC9248270 34087795

[26] Pessina F , Navarria P, Cozzi L, et al. Maximize surgical resection beyond contrast-enhancing boundaries in newly diagnosed glioblastoma multiforme: is it useful and safe? A single institution retrospective experience. J Neurooncol. 2017;135(1):129–139. 28689368 10.1007/s11060-017-2559-9

[27] Rahman M , Abbatematteo J, De Leo EK, et al. The effects of new or worsened postoperative neurological deficits on survival of patients with glioblastoma. J Neurosurg. 2017;127(1):123–131. 27689459 10.3171/2016.7.JNS16396

[28] Jakola AS , Gulati S, Weber C, Unsgård G, Solheim O. Postoperative deterioration in health related quality of life as predictor for survival in patients with glioblastoma: a prospective study. PLoS One. 2011;6(12):e28592. 22174842 10.1371/journal.pone.0028592 PMC3235141

[29] Lasocki A , Gaillard F, Tacey M, Drummond K, Stuckey S. Incidence and prognostic significance of non-enhancing cortical signal abnormality in glioblastoma. J Medical Imag Radiat Oncol. 2016;60(1):66–73. 10.1111/1754-9485.12421 26597591

[30] Niyazi M , Andratschke N, Bendszus M, et al. ESTRO-EANO guideline on target delineation and radiotherapy details for glioblastoma. Radiother Oncol. 2023;184:109663–109673. 37059335 10.1016/j.radonc.2023.109663

[31] Lasocki A , Gaillard F. Non-contrast-enhancing tumor: a new frontier in glioblastoma research. AJNR Am J Neuroradiol. 2019;40(5):758–765. 30948373 10.3174/ajnr.A6025 PMC7053910

[32] Lawrence YR , Li XA, el Naqa I, et al. Radiation dose-volume effects in the brain. Int J Radiat Oncol Biol Phys. 2010;76(3 Suppl):S20–S27. 20171513 10.1016/j.ijrobp.2009.02.091 PMC3554255

[33] Gondi V , Tomé WA, Mehta MP. Why avoid the hippocampus? A comprehensive review. Radiother Oncol. 2010;97(3):370–376. 20970214 10.1016/j.radonc.2010.09.013 PMC2997490

[34] Goda JS , Dutta D, Krishna U, et al. Hippocampal radiotherapy dose constraints for predicting long-term neurocognitive outcomes: mature data from a prospective trial in young patients with brain tumors. Neuro-Oncol. 2020;22(11):1677–1685. 32227185 10.1093/neuonc/noaa076 PMC7690355

[35] Gebhardt BJ , Dobelbower MC, Ennis WH, et al. Patterns of failure for glioblastoma multiforme following limited-margin radiation and concurrent temozolomide. Radiat Oncol (London, England). 2014;9(1):130. 10.1186/1748-717X-9-130 PMC4055938 24906388

[36] Azoulay M , Chang SD, Gibbs IC, et al. A phase I/II trial of 5-fraction stereotactic radiosurgery with 5-mm margins with concurrent temozolomide in newly diagnosed glioblastoma: primary outcomes. Neuro-Oncol. 2020;22(8):1182–1189. 32002547 10.1093/neuonc/noaa019 PMC7594571

[37] Guram K , Smith M, Ginader T, et al. Using smaller-than-standard radiation treatment margins does not change survival outcomes in patients with high-grade gliomas. Pract Radiat Oncol. 2019;9(1):16–23. 30195927 10.1016/j.prro.2018.06.001 PMC6487873

[38] Paulsson AK , McMullen KP, Peiffer AM, et al. Limited margins using modern radiotherapy techniques does not increase marginal failure rate of glioblastoma. Am J Clin Oncol. 2014;37(2):177–181. 23211224 10.1097/COC.0b013e318271ae03 PMC4485493

[39] Minniti G , Tini P, Giraffa M, et al. Feasibility of clinical target volume reduction for glioblastoma treated with standard chemoradiation based on patterns of failure analysis. Radiother Oncol J Eur Soc Ther Radiol Oncol. 2023;181:109435. 10.1016/j.radonc.2022.11.024 36529439

[40] Langhans M , Popp I, Grosu AL, et al. Recurrence analysis of glioblastoma cases based on distance and dose information. Radiother Oncol. 2023;183:109600. 36889597 10.1016/j.radonc.2023.109600 PMC10239332

[41] Lee SW , Fraass BA, Marsh LH, et al. Patterns of failure following high-dose 3-D conformal radiotherapy for high-grade astrocytomas: a quantitative dosimetric study. Int J Radiat Oncol Biol Phys. 1999;43(1):79–88. 9989517 10.1016/s0360-3016(98)00266-1

[42] Chang EL , Akyurek S, Avalos T, et al. Evaluation of peritumoral edema in the delineation of radiotherapy clinical target volumes for glioblastoma. Int J Radiat Oncol Biol Phys. 2007;68(1):144–150. 17306935 10.1016/j.ijrobp.2006.12.009

[43] Chamberlain MC. Radiographic patterns of relapse in glioblastoma. J Neurooncol. 2011;101(2):319–323. 21052776 10.1007/s11060-010-0251-4

[44] Chan JL , Lee SW, Fraass BA, et al. Survival and failure patterns of high-grade gliomas after two-dimensional conformal radiotherapy. J Clin Oncol. 2002;20(6):1635–1642. 11896114 10.1200/JCO.2002.20.6.1635

[45] Elson A , Bovi J, Siker M, Schultz C, Paulson E. Evaluation of absolute and normalized apparent diffusion coefficient (ADC) values within the post-operative T2/FLAIR volume as adverse prognostic indicators in glioblastoma. J Neurooncol. 2015;122(3):549–558. 25700835 10.1007/s11060-015-1743-z

[46] Niyazi M , Geisler J, Siefert A, et al. FET–PET for malignant glioma treatment planning. Radiother Oncol. 2011;99(1):44–48. 21458093 10.1016/j.radonc.2011.03.001

[47] Ladefoged CN , Law I, Anazodo U, et al. A multi-centre evaluation of eleven clinically feasible brain PET/MRI attenuation correction techniques using a large cohort of patients. Neuroimage. 2017;147:346–359. 27988322 10.1016/j.neuroimage.2016.12.010 PMC6818242

